# Effect of Polysaccharides from *Bletilla striata* on the Healing of Dermal Wounds in Mice

**DOI:** 10.1155/2019/9212314

**Published:** 2019-10-24

**Authors:** Chen Zhang, Yanan He, Zhejie Chen, Jinfeng Shi, Yan Qu, Jinming Zhang

**Affiliations:** ^1^Pharmacy College, Chengdu University of Traditional Chinese Medicine, Chengdu 611137, China; ^2^State Key Laboratory of Quality Research in Chinese Medicine and Institute of Chinese Medical Sciences, University of Macau, Taipa, Zhuhai, Macao SAR, China

## Abstract

*Bletilla striata* has been largely used in traditional folk medicine in China as a wound healing agent and to treat gastritis and several other health problems. Some studies have shown that plant polysaccharides may have the ability to promote wound healing. The aim of this work was to evaluate the wound healing activity of the polysaccharide extracted from *Bletilla striata.* Firstly, a *Bletilla striata* polysaccharide was extracted by water extraction and alcohol precipitation and characterized by Fourier transform infrared spectroscopy. The *Bletilla striata* polysaccharide was then tested for cell migration and proliferation using the mouse fibroblast cell line. Then, the *Bletilla striata* hydrogel was fabricated for acute wound health care of the mouse full-thickness excision. The results showed that the BSP enhanced the proliferation and migration of L929 cells. The superior wound healing capacity of the BSP hydrogel was demonstrated that it significantly accelerated the wound healing process *in vivo* in full-thickness skin defect wounded models. Compared to the saline group, the BSP hydrogel could accelerate wound healing and promote re-epithelialization and collagen deposition by means of TGF-*β*/Smad signal pathway activation. Taken together, BSP hydrogel would be a useful pharmaceutic candidate for acute cutaneous wound health care.

## 1. Introduction

Wound healing involves four overlapping phases of coagulation, inflammation, proliferation, and tissue remodeling and is a dynamic and complex process controlled by many factors [1]. The way and time of wound healing depends on the degree of injury, tissue regeneration ability, necrotic tissue, foreign body infection, and so on [2]. With the in-depth study of wound healing process in recent years, various strategies have been developed to attain skin lesion closure, including antibacterial ointments containing enzymatic substances, synthetic growth factors, polyurethane, hyaluronic acid hydrogels, and alginate fiber dressings [3]. However, the ideal curative features such as efficacy in absorbing wound exudates, flexibility, durability and adherence, and low cost are not fully attained yet [4, 5].

Natural products from plants and marine lives, as an alternative source of drugs which modulate the inflammatory process in a shorter time period and promote skin tissue regeneration in the treatment of skin lesions, have become a popular topic of scientific research, such as honey [6], propolis [7], marine fungus [8], squid ink polysaccharide [9], and so on. These natural products are believed to offer effective, affordable, and accessible forms of treatment. Among these natural products, polysaccharides have attracted widespread attention and have been demonstrated to promote skin tissue repair on the healing of dermal wounds by ameliorating oxidative stress, inflammation, and secondary trauma [10].

*Bletilla striata* polysaccharide (BSP) is a water-soluble polysaccharide extracted from the plant *Bletilla striata* and it is a polymer consisting of several 1,4-chain mannitol residues and 1,4-chain glucose residues [11, 12]. The BSP has good pharmacological activities, such as promoting wound healing, antibacterial, antitumor, antifibrotic, and so on [13, 14]. Simultaneously, the BSP has great potential and application prospects in biological delivery systems, wound dressings, and other biomaterials because of its great biodegradability and biocompatibility [15–17]. In our previous study, the BSP has been found to play a nontrivial role in promoting oral ulcer healing as a component in buccoadhesive wafers [11]. Nevertheless, the promoting wound healing effect of the BSP on dermal wound injury and its related mechanisms remain largely unclear.

In this study, we extracted the BSP by the method of water extraction and alcohol precipitation. Then the wound scratch and cytotoxicity assays were carried out to ensure the BSP's efficacy and toxicity profiles. Furthermore, according to the properties of the BSP, the polysaccharide hydrogels were prepared to repair dermal wounds in mice. In consideration of cut as the most common acute wound, murine full-excisional skin wound was employed to evaluate the wound healing effect of the BSP hydrogel. Meanwhile, the potential healing promoting mechanism of the BSP *in vivo* were also explored. In the light of these results, the present study wound provide a useful and efficient dosage form for topical wound healing of the BSP for the first time. We hope to develop a natural plant polysaccharide hydrogel product for promoting skin wound healing through our research.

## 2. Materials and Methods

### 2.1. Materials

The root of *Bletilla striata* (Thunb.) Reichb. F. was purchased from Sichuan New Lotus Traditional Chinese Medicine Pieces Co., Ltd. (Chengdu, China). All samples were deposited at the Chengdu University of Traditional Chinese Medicine, Chengdu, China.

The quantitative kits for SOD and iNOS by the spectrophotometric method were purchased from Jiancheng Bioengineering Institute (Nanjing, China). The enzyme-linked immunosorbent assay (ELISA) kits for tumor necrosis factor-alpha (TNF-*α*) and interleukin-1*β* (IL-1*β*) were supplied by MultiScience Lianke Biotech Co., Ltd. (Hangzhou, China). Methyl thiazolyl tetrazolium (MTT) was purchased from Dalian Meilun Biotechnology Co., Ltd. (Dalian, China).

### 2.2. Preparation of BSP

The BSP was prepared as described previously [18]. In brief, the dry root of *Bletilla striata* was homogenized and dispersed in boiled water for 4 h. After removal of impurities by filtration, the possible polysaccharide fractions were precipitated by three volumes of cold ethanol (4°C) overnight. The precipitate was repeatedly washed with ethanol solution (v/v: 95%), resuspended in distilled water, and added with Sevag's solution (1/3 volume chloroform/*n*-butanol (4 : 1, v/v)), followed by rigorous agitation to precipitate proteins. After centrifugation, the aqueous phase was successively collected to repeat the Sevag deproteinisation process until no obvious denatured protein precipitate was found in the n-butanol interlayer. The deproteinated aqueous fraction was dialyzed against the membrane with molecular weight cut-off (MWCO) of 5 KDa. The resultant liquor was precipitated with 3 times the volume of 95% ethanol and placed in 4°C for 24 h. Precipitate of the polysaccharides was collected by centrifugation at 5000*g* for 10 min. The precipitate was washed with 95% ethanol and water-free ethanol, respectively, after suction and lyophilized in vacuo.

### 2.3. Physicochemical Characterization of BSP

#### 2.3.1. Attenuated Total Reflection Fourier Transform Infrared Spectroscopy (ATR-IR)

The ATR-IR spectrometer of the polysaccharides used was a Nicolet 460 FT-IR with Smart Golden Gate Diamond ATR (Thermo Scientific, Germany). About 3–5 mg of powdered sample was placed on the sample holder and compressed under pressure to form a pellet. The Spectrums of the powdered form of the BSP were measured in the wave number range of 500–4000 cm^−1^ using 32 scans.

#### 2.3.2. Thermogravimetric-Differential Thermal Analysis (TG-DTA)

TG-DTA of the BSP powder was carried out simultaneously using a NETZSCH simultaneous thermal analyzer STA 449F3 Jupiter equipped with a TG-DTA sample carrier type supporting a ptRh10-Pt thermocouple (Netzsch, Germany). TG-DTA thermograms were taken using a standard Al_2_O_3_ pan. Nitrogen was used as a sweeping gas, and the heating rate was 20°C/min. The polysaccharide sample (20 mg) was loaded in a pan without further treatment. The initial and end temperatures are 25°C and 500°C, respectively.

### 2.4. *In Vitro* Cell Culture Assays

Mouse fibroblast cell line (L929; CCL-1, Mus musculus) purchased from the American Type Cell culture/ATCC was cultivated in DMEM with 10% fetal bovine serum and 1% penicillin streptomycin at 37°C in a humidified incubator with 5% carbon dioxide.

### 2.5. MTT Cell Proliferation Assays

The cell proliferation induced by the BSP was determined using a tetrazolium salt (MTT) assay *in vitro*. Briefly, cells were seeded in a volume of 200 *μ*L (3000 cells/well) on 96-well plates after cultivation with different concentrations of BSP. The culture medium containing serum was replaced by MTT every 24 h. A final MTT concentration of 0.5 mg/mL was added to the wells followed by incubation for 4 h at 37°C. The supernatant was discarded and replaced with DMSO (150 *μ*L/well). The optical densities (OD) were measured at 570 nm with a microplate reader. The experiment was repeated in triplicate. The viable concentration was calculated using GraphPad Prism 5.0.

### 2.6. Wound Scratch Assay

The assay was conducted as described by the literature method with slight modifications [19]. The wound healing assay mimics the proliferation and migration of cells during wound injury *in vivo*. It serves as an excellent *in vitro* assay for the study of cell-cell or cell-matrix interactions in cellular proliferation and migration to initiate the wound repair process. L929 cells were seeded in the 6-well plate, and 10% FBS growth medium containing the serum-free medium supplemented with the BSP was grown to 90% confluence. After treatment for 48 h, the culture medium was removed and the monolayers were scratched using a 200 *μ*L pipette to create a uniform cell-free wound area. Debris was removed by gently washing with sterile PBS. Cell movement into the wound area was monitored and photographed at 0, 24, and 48 h using an optical microscope.

### 2.7. Animal Experimental Protocol

Healthy adult male Kunming mice (18∼22 g) were maintained individually in the IVC mice cages, in a temperature-controlled room (25°C) under a 12-h light and 12-h dark cycle, to avoid the bacteria interference. The animal experiments were performed in compliance with the experimental protocols approved by the Animal Investigation Committee of the Institute of Pharmacy College of Chengdu University of TCM.

### 2.8. *In Vivo* Wound Healing Assay

#### 2.8.1. Preparation and Structural Properties of the BSP Hydrogel

The lyophilized BSP were dissolved in distilled water by magnetic stirring for 60 min, then swelled overnight at 4°C, and finally prepared into BSP solutions of different concentrations (10, 20, and 40 mg/mL). The cross-section of the hydrogel was examined using scanning electron microscopy (SEM; ZEISS SUPRA 40, Germany) operated at 20 kV acceleration voltage. Before observation, the hydrogels were freeze-dried at −80°C for 2 days, fractured using a blade to obtain the hydrogel sheet, and Au sputter coated with ∼30 nm coating layer.

#### 2.8.2. Excision Animal Model

All mice were anesthetized by IP. injection 2% pentobarbital (45 mg/kg body weight), and the dorsal hair was shaved using a shaving machine. The surgical area was disinfected with Betadine. Full-thickness excisional wounds were created using 8 mm biopsy punch modified tools in the dorsal site of Kunming mice. Animals were divided into two groups (20 mice per group). The BSP hydrogel was applied to cover the wound area every day for 12 days, while the control group received no treatment (dose of BSP 0.4 g/Kg). The kinetics of wound closure was treated through digital photography, and wound area and percentage of wound closure were measured immediately after wounding and every day until 12 days using the following equation [20]:

(1) $$\begin{array}{c} \text{wound closure}\left(\%\right)=\left[\frac{\left(A_{O}-A_{n}\right)}{A_{O}}\right]\times 100, \end{array}$$

where *A*_*O*_ was the original wound area after surgery and *A*_*n*_ was the wound area on day *n* after wounding.

At day 6 and 12, all the BSP dressings were removed, and animals were scarified under anesthesia. Tissue specimens of the incised skin in each group were collected for histopathological examinations and biochemical analysis.

#### 2.8.3. Serum Biochemical Factor Determination

At day 12, all the rest of mice were sacrificed by overdose anesthetics. Serum samples were obtained by blood centrifugation at a rotational speed of 3500/min for 10 min. Cytokines in serum samples including TNF-*α*, IL-1*β*, iNOS, and SOD were measured using various test kits. All procedures were according to the instructions of the manufacturer.

#### 2.8.4. Histological Analysis of Wound Healing

One-third of mice in each group were euthanized and sacrificed on day 6 and day 12 post injury. Wound lesion tissues were excised, fixed overnight in 4% buffered formalin solution, and embedded in paraffin. Tissue sections (5 mm) were stained with hematoxylin and eosin (H&E) for morphological assessment. The collagen analysis of skin wounds was performed using Masson's Trichrome Stain Kit (Sigma) and Siriusred staining.

#### 2.8.5. Related Gene Expression by RT-PCR Analysis

The TGF-*β*/Smad pathway was involved in collagen deposition, which was an important step in wound healing [21, 22]. The expression levels of three genes, i.e., TGF-*β*1, Smad2, and Smad4, were analyzed in the collected wound lesion on day 6 and day 12 by RT-PCR technology. The specific operation is as follows: firstly, total RNA in tissues were isolated using TRIzol™ Reagent (Invitrogen Co., Ltd., California, USA). RNA samples were subjected to DNase I treatment to remove genomic DNA contamination in the presence of RNase inhibitor. Then, the quality and quantity of RNA were checked by RNA gel electrophoresis and ultramicrospectrophotometer (Thermo Fisher, model: NanoDrop2000, USA). Total RNA (2 *μ*g) was reverse transcribed to cDNA using RevertAi™ M-MuLV reverse transcriptase and oligo (dT)_18_ (Thermo Fisher, USA) by incubating the reaction mixture at 42°C for 60 min and 60°C for 5 min. The resulting first-strand cDNA was used as the template in the semiquantitative polymerase chain reaction (PCR). The cDNA product was subjected to PCR (apo. protect, eppendorf) for 40 cycles by gene specific primers. The primers used to amplify cDNA by PCR were designed by Primer Express software: TGF-*β*1 (F5′- AGGGCTACCATGCCAACTTC-3′, R5′-CCACGTAGTAGACGATGGGC-3′), Smad2 (F5′-TAGGTGGGGAAGTGTTTGCTGA-3′, R5′-TGACAGACTGAGCCAGAAGAGC-3′), Smad4 (F5′-CCAGGCAGAGCATCAAGGAA-3′, R5′-TCAGTCTAAAGGCTGTGGGTCC-3′), and GAPDH (F5′-CCTCGTCCCGTAGACAAAATG-3′, R5′-TGAGGTCAATGAAGGGGTCGT-3′). PCR products were determined by fluorescence quantitative PCR equipment (ABI, model: StepOne plus, USA). All samples were analyzed in triplicates.

### 2.9. Statistical Analysis

Data were expressed as mean value ± standard error. All the data were statistically analyzed by one-way analysis of variance (ANOVA) using IBM SPSS Statistics analysis of variance (20.0) and GraphPad Prism software. Statistical difference was considered significant when probability values were less than 0.05 (*p* < 0.05).

## 3. Results and Discussion

### 3.1. Preparation and Characterization of the BSP Hydrogel

The polysaccharide content (65.3%) in the extracts was determined using the phenol-sulfuric acid method. The ability of *Bletilla striata* polysaccharides to form hydrogels is one of its important characteristics, and according to our preliminary experiments, BSP cannot form hydrogels at low concentrations (10 mg/mL and 20 mg/mL). However, concentration of 40 mg/mL of BSP water solution can form the hydrogel through water swelling. Eventually, we employed concentration of the BSP (40 mg/mL) in this study.

**Figure 1:**
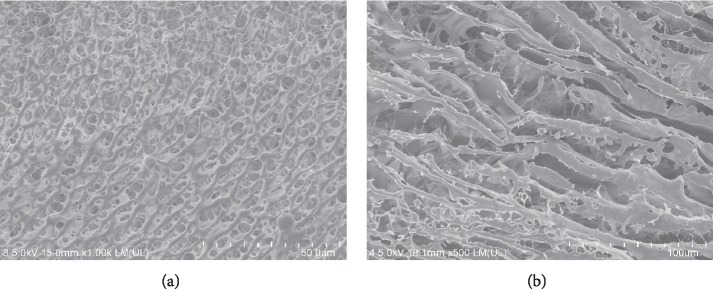
SEM micrographs of the lyophilized BSP hydrogel in cross-sectional view at magnification of × 100 (a) and × 500 (b).

SEM was used to evaluate the topographic characteristics and morphology of the BSP hydrogel as they are related to swelling, dissolution, and release characteristics. Representative SEM images of the lyophilized BSP hydrogel are shown in Figure 1(a) and 1(b). Micrographs of the BSP hydrogel presented a porous interconnecting network. Some pores formed continuous channels, while others exhibited overlapping sheet-like structures. This porous structure would provide a large surface area to accelerate the process of swelling and dissolution.

### 3.2. ATR-IR Analysis

**Figure 2:**
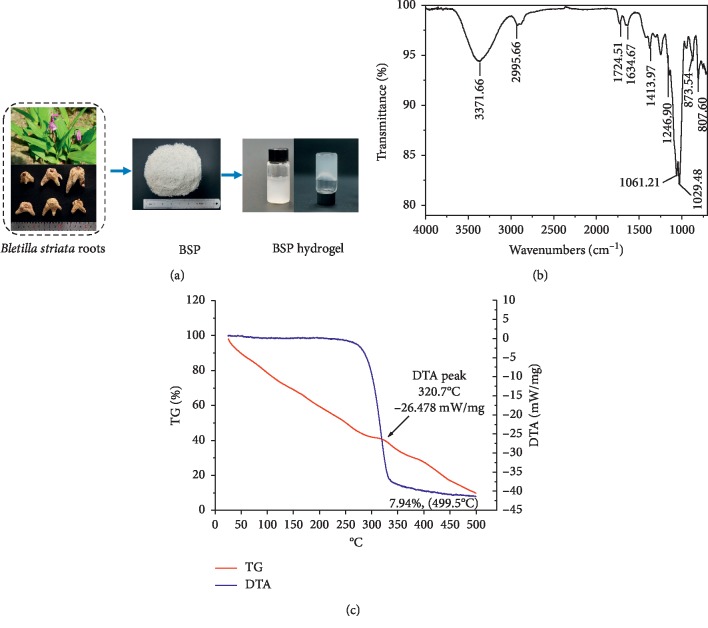
(a) Photographic appearance of the BSP and BSP hydrogel. (b) FT-IR spectra of the BSP showing the various absorption peaks. (c) TG-DTA weight loss curve of the BSP under inert atmosphere at a heating rate of 10°C/min.

The ATR-IR spectra of the BSP is shown in Figure 2(b). The two absorptions in the region 3600–2800 cm^−1^ were due to stretching vibration of C-H and O-H, respectively. The weak peak at 727.94 cm^−1^ indicated the existence of acetyl. The absorption at approximately 1650.8 cm^−1^ was attributed to the asymmetric stretching vibration of C=O absorption. A strong extensive band in the region of 1200–1000 cm^−1^ was caused by the stretching vibrations of C-OH side groups and the C-O-C glycosidic band vibrations, which is a characteristic band for each particular polysaccharide. Moreover, the only one peak at about 1041.39 cm^−1^ revealed the presence of a pyranose form, and the weak signal at 921.82 cm^−1^ represented the *β*-glycosidic linkage at 863.97 cm^−1^ that evidenced the existence of D-mannopyranose units and at 806.11 cm^−1^ stood for mannose residues.

### 3.3. TGDTA Analysis

TGA was performed to study the decomposition pattern and the thermal transition occurring in the course of heating under inert atmosphere of polysaccharides as shown in Figure 2(c). Various thermal effects and enthalpy changes of the polysaccharide were exhibited, and an early endothermic event located between 90-100°C was attributed to water evaporation and loss of mass. The second weight loss region located between 290-330°C is attributed to the degradation and thermal decomposition of the polysaccharide. The third weight loss event is located in the range of 340–460 due to the oxidation of organic matter.

### 3.4. Effect of BSP on Cell Proliferation *In Vitro*

**Figure 3:**
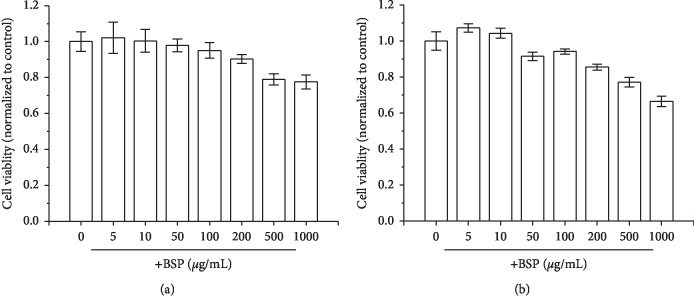
Effects of various concentrations of the BSP on cell proliferation of L929 cells after 24 h (a) and 48 h (b) treatment (mean ± SD, *n* = 3).

MTT cell proliferation assay was employed to detect the cytotoxicity of the BSP on L929 cells. Even when 100 *μ*g/mL BSP was given, there was no significant effect on viability of L929 cells both in 24 h and 48 h treatment (Figure 3). Notably, 5 *μ*g/mL and 10 *μ*g/mL of the BSP could slightly promote cell growth, compared with the control group. These results demonstrated the noncytotoxicity of the BSP on gastric mucosa epithelial cells.

### 3.5. Wound Scratch Assay

**Figure 4:**
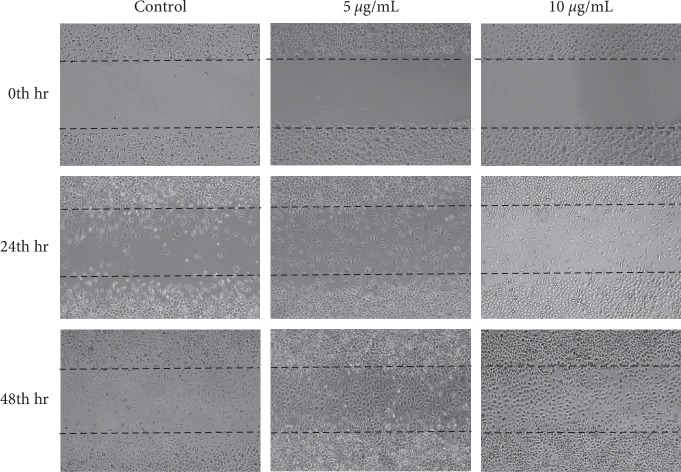
Wound healing assay using L929 cells treated with the BSP at concentrations of 5 *μ*g/mL and 10 *μ*g/mL (mean ± SD, *n* = 3).

To determine whether the BSP promoted L929 cell migration in skin, we performed wound healing assays. At 0 h, the cell scratch spacing of the control group, 5 *μ*g/mL group, and 10 *μ*g/mL group was basically the same. At 24 h and 48 h, the cell scratch spacing of each group became narrower. Scratches even disappeared at 48 h in 5 *μ*g/mL and 10 *μ*g/mL groups. The cell scratch test showed that the BSP could significantly improve the migration of L929 cells and facilitate wound healing (Figure 4).

### 3.6. Accelerating Full-Thickness Excision Wound Healing

**Figure 5:**
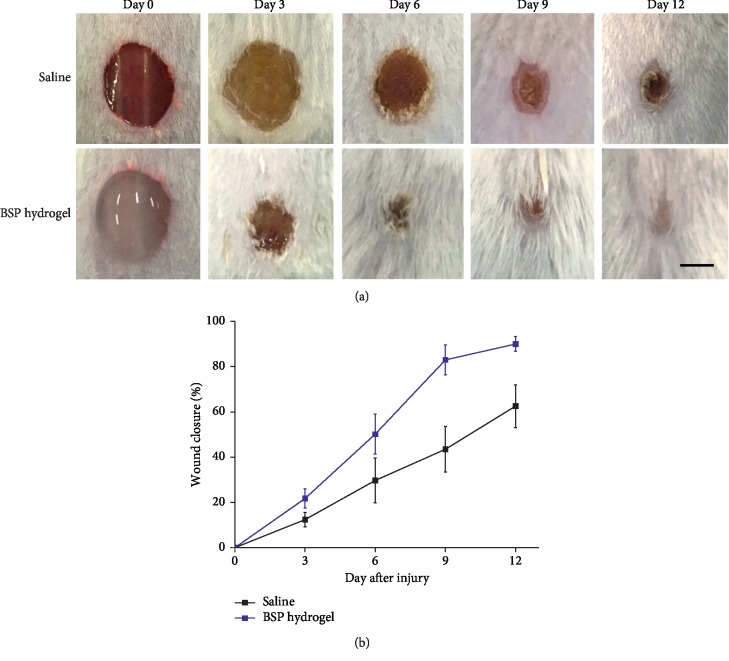
*In vivo* wound healing effect of the BSP hydrogel on the full-thickness excision wound model. (a) Wound photographs at 0, 3, 6, 9, and 12 days post injury in saline, the BSP hydrogel group. The scale bar indicates 5 mm. (b) Wound closure rate on wound area at 0, 3, 6, 9, and 12 days.

The wound healing benefit activity of the BSP hydrogel was evaluated by a full-thickness excision wound model (Figure 5(a)). Compared to the control group, the BSP hydrogel could promote the healing process after wound induction. It would be attributed to the favorable water absorption ability and water vapor permeability of the hydrogel to maintain the moisture in the environment in wound tissue. On days 3 and 6, the unhealed area of the BSP hydrogel group was smaller than that of the control groups (Figure 5(b)). Only the BSP hydrogel achieved wound closure within 12 days, indicating that the BSP hydrogel possessed a more efficient healing effect on full-thickness wounds (Figure 5(a)).

**Figure 6:**
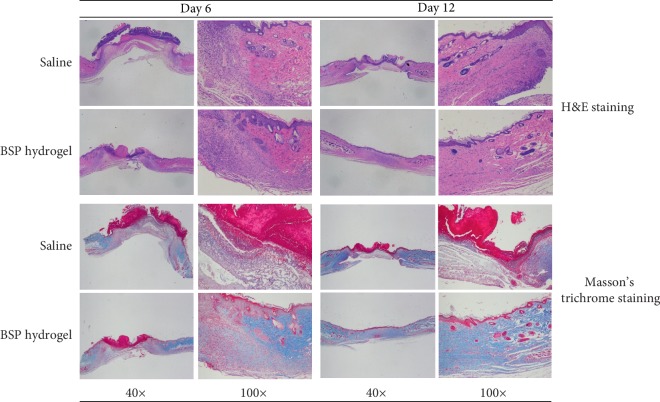
The effect of the BSP hydrogel on epithelium proliferation and collagen deposition in full-thickness excision wound by H&E and Masson's trichrome staining.

To better understand the effect of BSP hydrogel treatment on wound healing, the epidermal migration and collagen deposition, which are the most common indications in wound healing process [23], at full-thickness excision wound was analyzed by Masson's trichrome staining [24]. The results showed that the thickness of the epidermis in the BSP hydrogel group remarkably increased than that of the control group at day 12, and the length of the newly formed epithelium in the BSP hydrogel treated group was significantly longer than that in the control group at day 6 and 12, respectively (Figure 6). Furthermore, the collagen fibers in the BSP hydrogel were more extensive and orderly arranged than those in untreated control groups, showing more blue area at either day 6 or day 12. Also, the collagen deposition at wounds showed the time-dependent manner, in which the treated groups at day 12 displayed more collagen levels.

**Figure 7:**
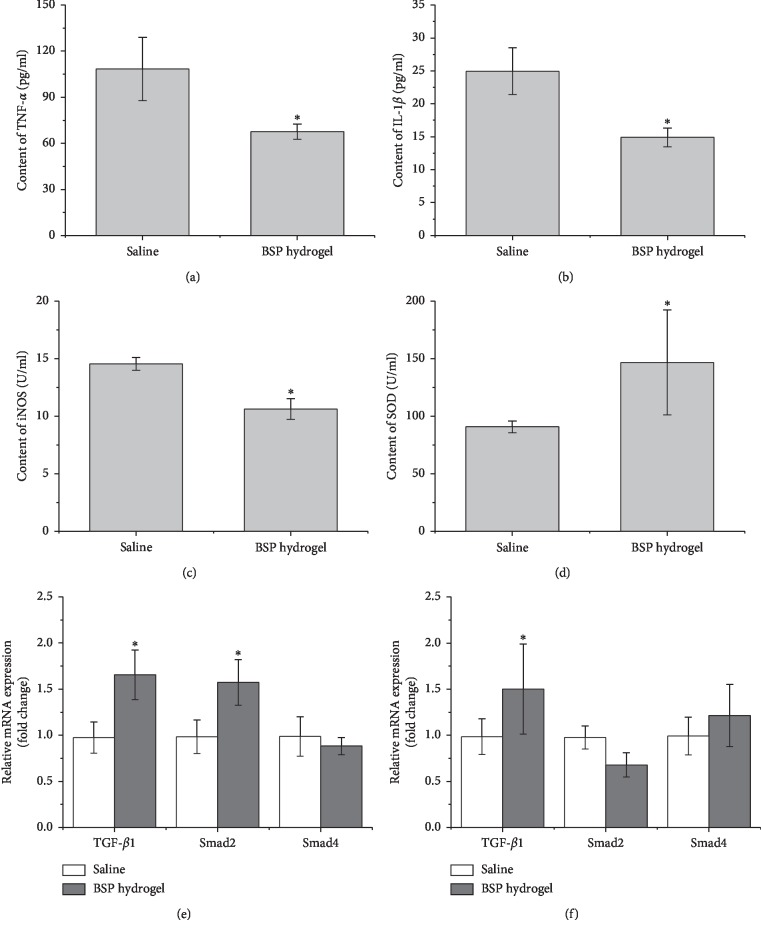
The levels of TNF-*α* (a), IL-1*β* (b), iNOS (c), and SOD (d) of the serum in the saline control and the BSP hydrogel group at 12 days after injury using test kits. Quantitative assessment of TGF-*β*, Smad2, and Smad4 mRNA expression in various groups at day 6 (e) and day 12 (f) postinjury wounding. Note: ^*∗*^*p* < 0.05 vs. saline-treated group.

The contents of TNF-*α*, IL-1*β*, iNOS, and SOD in mice tissues at day 12 post injury in various groups were compared. In accordance with the histomorphological results, mice treated by BSP hydrogel groups exhibited the fewest contents of inflammatory cytokines (TNF-*α*, IL-1*β*, and iNOS) compared to the control group (Figure 7(a)–7(c)). On the contrary, SOD as one of the body's primary internal antioxidant defenses plays a critical role in reducing internal inflammation and lessening pain associated with conditions [25]. BSP hydrogel treatment could significantly elevate the SOD production in wound tissue (Figure 7(d)). These results contributed to the optimal wound healing profiles in mice treated with the BSP hydrogel. At day 12 post injury, mice's wounds in the BSP hydrogel group have been almost healed up. Furthermore, the intracellular Smad protein is well known to transduce the extracellular TGF-*β* signal to the fibroblast nucleus for collagen production [26]. Thus, TGF-*β*/Smad signaling axis has been demonstrated the important role in collagen production [27]. Herein, mRNA levels of TGF-*β*1, Smad2, and Smad4 at day 6 and day 12 were quantitatively measured using RT-PCR analysis. The mRNA expression as densitometry band intensities of the target gene relative to GAPDH was shown in Figure 7(e) and 7(f). Results indicated that especially BSP hydrogel treatment exhibited the highest expression of TGF-*β*1and Smad2 at day 6, and the highest expression of TGF-*β*1at day 12. Therefore, these results indicated that the BSP hydrogel could increase the Smad2 heterocomplex translocation into the nucleus and induce the TGF-*β*-specific transcriptional response.

## 4. Conclusion

In this manuscript, the BSP, prepared by water extraction and alcohol precipitation, was found to be the most promising wound healing agent, as shown in the wound scratch assays. According to the properties of BSP, the BSP hydrogel was successfully fabricated for acute wound health care of full-thickness excision. The *in vivo* study demonstrated that the BSP as a wound healing patch significantly improved wound contraction, epithelization and the collagen deposition, and inflammatory response. Notably, to activate TGF-*β*1/Smad2 pathway was the related wound healing mechanism of the BSP hydrogel. Our results indicated that the BSP hydrogel can be a promising therapeutic approach for topical application in treatment of cutaneous wounds.

## Data Availability

The data used to support the findings of this study are available from the corresponding author upon request.
